# Analysis of the Technical Accuracy of a Patient-Specific Stereotaxy Platform for Brain Biopsy

**DOI:** 10.3390/jpm14020180

**Published:** 2024-02-05

**Authors:** Marcel Müller, Dirk Winkler, Robert Möbius, Michael Werner, Welf-Guntram Drossel, Erdem Güresir, Ronny Grunert

**Affiliations:** 1 Fraunhofer Institute for Machine Tools and Forming Technology, Nöthnitzer Straße 44, D-01187 Dresden, Germany; michael.werner@iwu.fraunhofer.de (M.W.); welf-guntram.drossel@iwu.fraunhofer.de (W.-G.D.); ronny.grunert@medizin.uni-leipzig.de (R.G.); 2 Department of Neurosurgery, Faculty of Medicine, University Clinic of Leipzig, Liebigstraße 20, D-04103 Leipzig, Germany; dirk.winkler@medizin.uni-leipzig.de (D.W.); robert.moebius@medizin.uni-leipzig.de (R.M.); erdem.gueresir@medizin.uni-leipzig.de (E.G.)

**Keywords:** brain biopsy, stereotaxy, 3D printing, intracranial, patient-specific, accuracy

## Abstract

The use of stereotactic frames is a common practice in neurosurgical interventions such as brain biopsy and deep brain stimulation. However, conventional stereotactic frames have been shown to require modification and adaptation regarding patient and surgeon comfort as well as the increasing demand for individualized medical treatment. To meet these requirements for carrying out state-of-the-art neurosurgery, a 3D print-based, patient-specific stereotactic system was developed and examined for technical accuracy. Sixteen patient-specific frames, each with two target points, were additively manufactured from PA12 using the Multi Jet Fusion process. The 32 target points aim to maximize the variability of biopsy targets and depths for tissue sample retrieval in the brain. Following manufacturing, the frames were measured three-dimensionally using an optical scanner. The frames underwent an autoclave sterilization process prior to rescanning. The scan-generated models were compared with the planned CAD models and the deviation of the planned target points in the XY-plane, Z-direction and in the resulting direction were determined. Significantly lower (*p* < 0.01) deviations were observed when comparing CAD vs. print and print vs. sterile in the Z-direction (0.17 mm and 0.06 mm, respectively) than in the XY-plane (0.46 mm and 0.16 mm, respectively). The resulting target point deviation (0.51 mm) and the XY-plane (0.46 mm) are significantly higher (*p* < 0.01) in the CAD vs. print comparison than in the print vs. sterile comparison (0.18 mm and 0.16 mm, respectively). On average, the results from the 32 target positions examined exceeded the clinically required accuracy for a brain biopsy (2 mm) by more than four times. The patient-specific stereotaxic frames meet the requirements of modern neurosurgical navigation and make no compromises when it comes to accuracy. In addition, the material is suitable for autoclave sterilization due to resistance to distortion.

## 1. Introduction

A range of therapeutic options are available for treating intracranial lesions. Knowing the correct diagnosis is crucial in this regard. The interpretation of neuroradiological findings (using CT, MRI, angiography, PET, SPECT) does not currently allow a clear diagnosis to be made, and in very few cases does it justify further treatment, such as chemotherapy, radiation, systemic antibiotic therapy or surgical removal. Therefore, targeted therapy for unexplained intracranial lesions requires a local tissue sample to be removed as precisely as possible for further histopathological analysis.

The biopsy of an intracranial finding requires the application of a highly accurate stereotactic navigation method capable of intraoperative measurements in the millimeter range. Frameless and frame-based stereotactic systems are available on the market for this purpose. Among the most accurate systems are the frame-based systems from Leksell (Elekta, Stockholm, Sweden) and Brown-Roberts-Wells (Radionics, Burlington, VT, USA) with an error of 1–2 mm [1,2]. However, one downside of these systems is the bulky nature of the frames, which often represents a physical obstruction for patients and surgeons. A study that analyzed 89 samples from brain biopsies indicated that a frameless stereotactic brain biopsy is as effective as a frame-based one. A diagnosis was established in 88.9–99.7% of cases. However, the study showed that the diagnostic yield was associated with the size of the lesion. Lesions greater than 30 mm in diameter were associated with a higher rate of positive biopsy results (*p* = 0.001), whereas lesions less than 30 mm had postoperative intracerebral hematoma complications (*p* = 0.01) [3]. Between March 2013 and April 2018, more than 100 biopsies were performed using a frameless neuromate^®^ robot (Renishaw, Wotton-under-Edge, UK). The frameless robot was shown to be a viable alternative to frame-based stereotaxies. The average surgery time was 40 min (10 min to position the device and 30 min to perform the biopsy) [4]. These results demonstrated that there are no significant differences in the diagnostic yield in terms of post-biopsy morbidity or mortality between frameless and frame-based stereotactic systems. The only difference that has been confirmed is time savings when using the frameless systems [5].

Additive manufacturing technologies, such as 3D printing, show strong potential for growth in the medical engineering sector. The benefits offered by this technology come into play, for instance, in prostheses or surgical instruments, especially in personalized medicine. In addition to the use of metallic materials, such as titanium for implants, increasingly suitable 3D printing plastics are available. These are primarily used to produce anatomical models for clinical training and preoperative planning processes, such as brain tumor resections, pulmonary angiograms, and laparoscopic liver resections [6,7,8,9]. To improve the operational handling of conventional frame-based stereotactic systems, micro-stereotactic frames, such as the “STarFix microTargeting Platform” (FHC Inc., Bowdoin, ME, USA), have been introduced. The patient-specific frame is made of polyamide 11 plastic and is manufactured by means of selective laser sintering. Initial studies involving 263 patients (284 DBS implants over 6 years) showed a clinical accuracy of 1.99 ± 0.9 mm for deep brain stimulation. Furthermore, the operation time could be reduced by two hours compared to using conventional stereotactic frames [10].

At present, frame-based stereotaxy is therefore likely the best procedure with regard to diagnostic possibilities, as well as surgery-related morbidity and mortality, and is not surpassed by any other method in terms of accuracy. However, frame-based stereotaxy needs to be modified and adapted in view of current demands for neurosurgical treatments. These requirements have arisen from new hygiene guidelines for sterilization, rapid availability, and the increasing individualization of medical treatment. Another relevant factor is convenience for both patients and surgeons. To meet these requirements for modern neurosurgery, a 3D print-based patient-specific stereotaxic system was developed in the current study, which tries to implement them without compromising on accuracy compared to previous systems. Standardized biopsy needles have an outer diameter of up to 2.5 mm, whereas smaller biopsy needles pose a lower risk of postoperative bleeding [11]. This provides the necessary accuracy below the needle diameter for stereotaxic systems. The aim of this article is to evaluate the technical accuracy of this patient-specific stereotactic system using navigated brain biopsy as an exemplary procedure. In particular, the influence of defects relating to the additive manufacturing processes and the subsequent sterilization process on the accuracy of patient-specific frames is considered. The aim of this article is to show whether additive manufacturing technology offers the possibility of producing patient-specific stereotactic frames and whether these are below the clinically allowed target point deviation.

## 2. Materials and Methods

**Figure 1:**
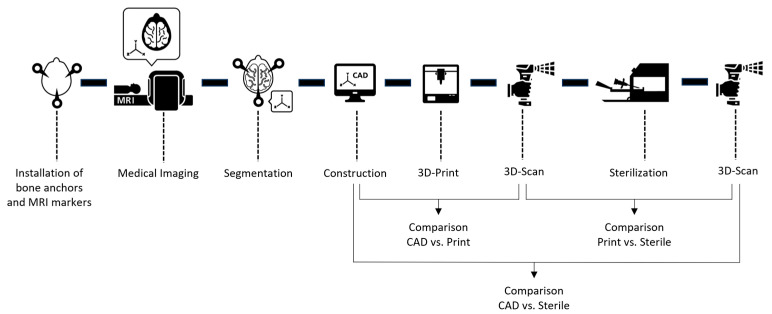
Process sequence of the applied method for accuracy analysis.

The methods described below are shown in Figure 1. The focus is on the 3D scan after manufacturing and after sterilization. The first steps of the process describe the procedure of constructing the patient-specific stereotactic frames (Section 2.1 and Section 2.2). In Section 2.3, the 3D scan measurement method and the measurement setup are described and in Section 2.4 the suitability of the measurement system is examined. The methodological comparison between the examination steps and the sterilization are described in Section 2.5 and Section 2.6.

### 2.1. Imaging and Design

Sixteen patient-specific stereotactic frames were designed and additively manufactured. Each frame was calibrated to focus on two defined target points in the cerebrum. T1-weighted magnetic resonance images (MRI volume dataset, 1 mm slices) were available as the initial image dataset to the surgeon for planning the target points.

**Figure 2:**
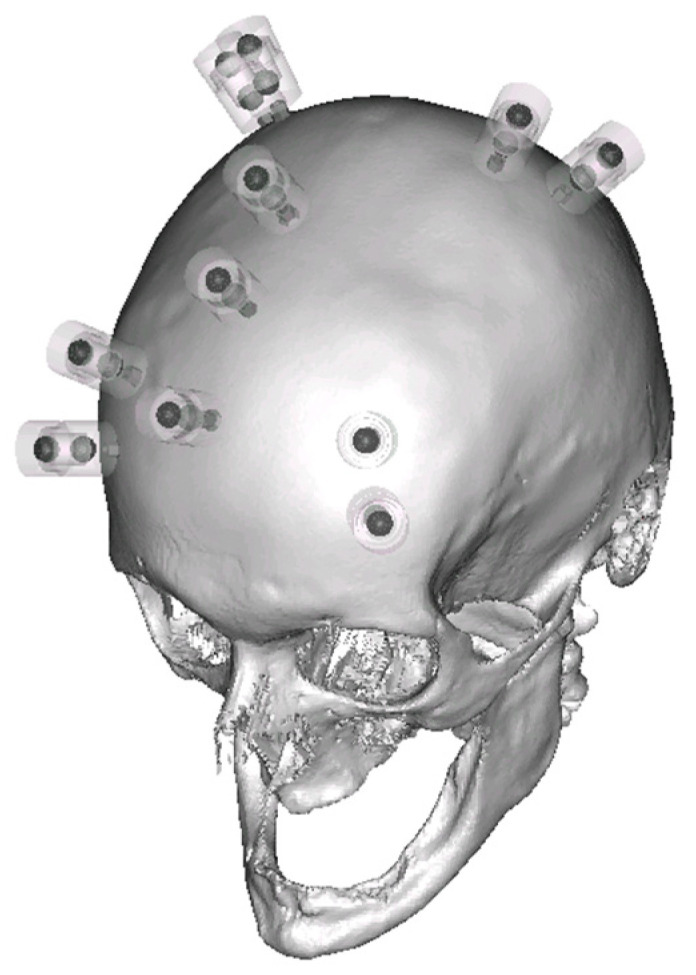
Eleven positions of the bone anchors and MRI markers on the skull.

Müller and colleagues showed the process steps from imaging to biopsy [12]. The image data were captured using a cadaver preparation of the human head (ethanol-fixed according to the in-house protocol of the Institute of Anatomy, Leipzig University). Bone anchors (type: 5 mm WayPoint, FHC Inc., Bowdoin, ME, USA) were applied to 11 positions in the frontal and occipital cranial dome (Figure 2).

To obtain the greatest possible range of variations in geometry, eight bone anchors were distributed in a quadrilateral with two bone anchors per corner. In addition, three bone anchors were arranged in the median plane in the AP direction. This placement allowed for any number of triangular geometries to be created for the frames. MRI markers were placed on the bone anchors prior to imaging. Unlike in Müller et al. 2019, each MRI marker was filled with two spherical vitamin D substrate capsules (Dekristol^®^ 20,000 IU, mibe GmbH Arzneimittel, Brehna, Germany) for referencing. The use of these capsules has been shown to produce excellent contrast during the MRI scan [13]. By capturing the image of two spheres, compared to one cylinder, the center of each sphere can be determined more accurately, regardless of the image-generating parameters (e.g., slice thickness, matrix size). It also enables an axis to be formed by the two centers, which precisely defines the alignment of the bone anchors.

The scan was performed using an Ingenia 3-Tesla MRI scanner (Philips, Hamburg, Germany), based on the imaging parameters presented in Table 1 (MRI parameters).

**Figure 3:**
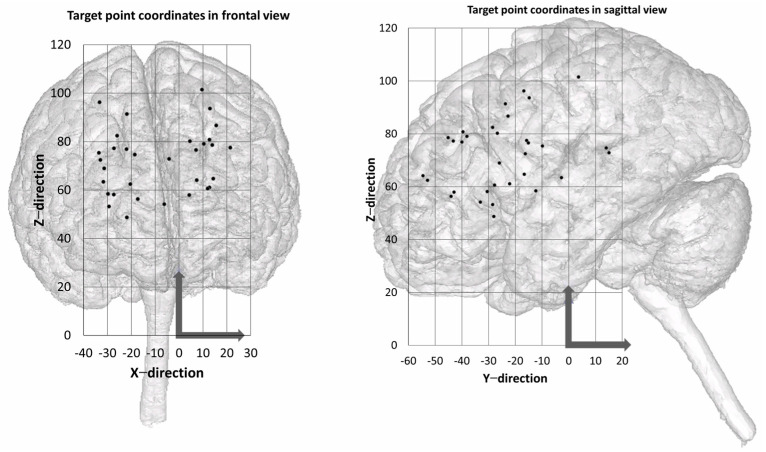
Location of target points in the cerebral brain in frontal view (**left**) and sagittal view (**right**). The coordinate origin and the positive axis direction are marked with an arrow.

**Figure 4:**
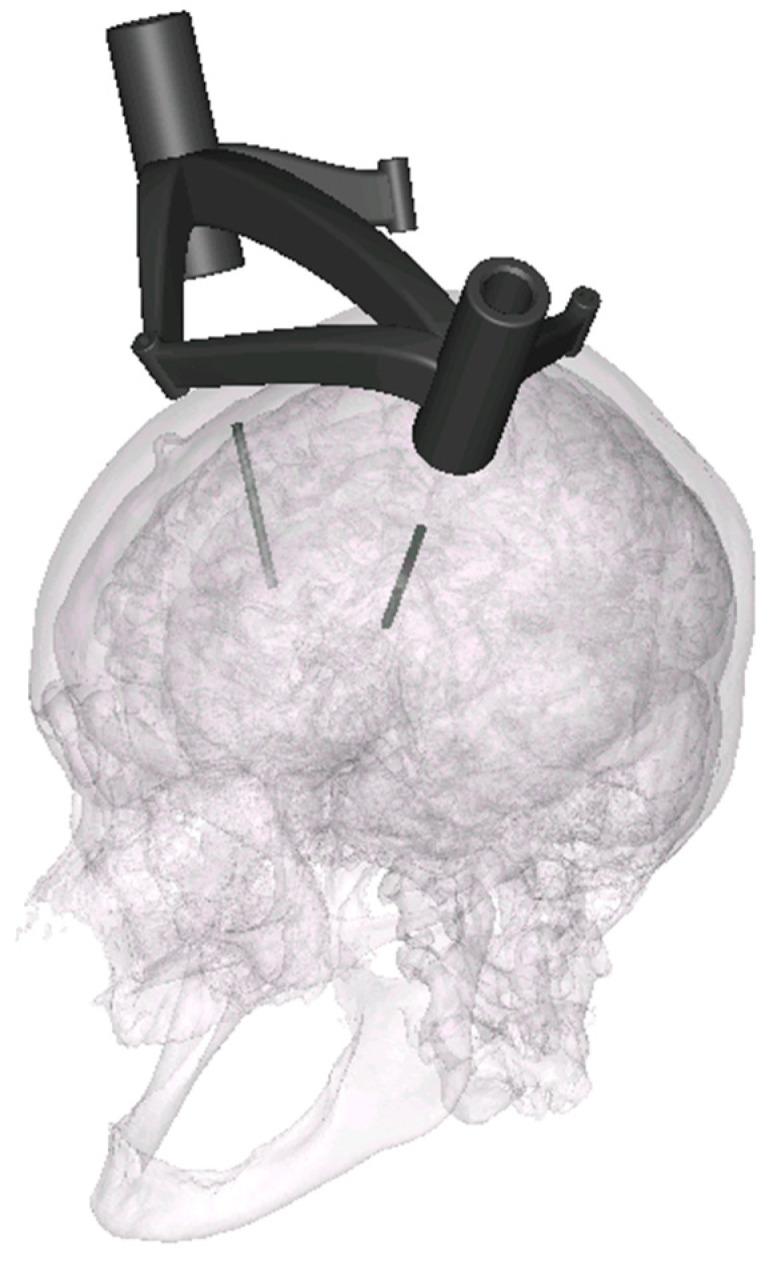
Constructed and placed biopsy frame on the skull with two target trajectories.

**Figure 5:**
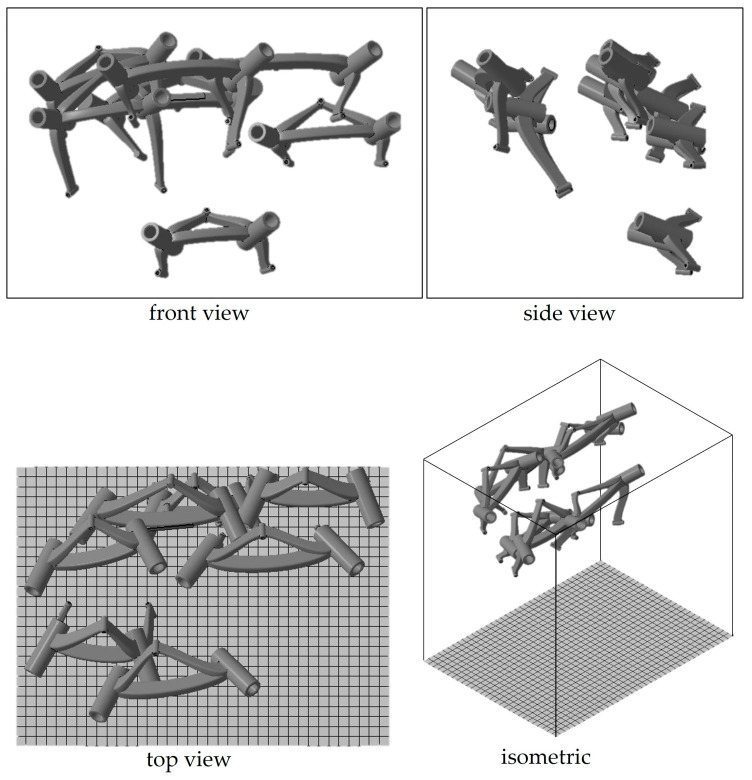
Alignment of the frames in the 3D printing build room.

Segmentation of the MRI markers and drawing of the biopsy needle trajectories were performed using the Mimics 16.0 program (Materialise, Leuven, Belgium). To increase the target points while maintaining the same frame production, two needle trajectories were planned for each frame at a different depth and in a different direction. Figure 3 visualizes the location of the target points. An experienced specialist in neurosurgery performed the positioning, with the aim of maximizing the variability of biopsy targets and depths in the brain. The total of 32 target points were homogeneously divided in a matrix comprising the distance between the target point and skullcap [distance A (<30, 30–40, 40–50, >50)] or skullcap and frame [distance B (<20, 20–25, 25–30, >30)] (data in millimeters). The center coordinates of the MRI markers—by fitting a sphere using the best-fit method—and the geometric trajectory points were calculated in GOM Inspect 2019 (Carl Zeiss GOM Metrology GmbH, Braunschweig, Germany). As in Müller et al. 2019, the coordinates were used to construct each frame using SolidWorks 2020 (Dassault Systèmes, Vélizy-Villacoublay, France). Each frame consisted of three fixing points and two guide points for the biopsy needles (Figure 4 and Figure 5).

### 2.2. Additive Manufacturing

The patient-specific stereotactic frames were developed using the Multi Jet Fusion additive manufacturing technology (Hewlett Packard, Palo Alto, CA, USA). This technology works with a powder-based fusing and detailing agent followed by heat exposure. The HP Jet Fusion 3D 4200 printer was used along with the plastic polyamide (PA12). The material is certified under USP Class I–VI and ISO 10993-5 and 10993-10 [14], and is therefore tested for in vitro cytotoxicity or irritation and skin sensitivity [15]. According to the technical data sheet, the additively manufactured PA12 has a tensile strength in the XY-plane of 1700 MPa and in the Z-direction of 1800 MPa. The elongation at break is 20% (XY-plane) and 15% (Z-direction). The heat deflection temperature (0.45 MPa) is 175 °C [16]. The material properties exhibit isotropic behavior and are approximately in the range of a commercially available PA12 (tensile strength: 1100–1800 MPa, elongation at break: 250–280%, heat deflection temperature: 118 °C) [17]. The additively processed PA12 is therefore very well suited to manufacturing medical technology products. The benefits offered by this technology are twofold. First, no support structure is necessary when producing the components. Second, the slice height in the Z-direction is low with the HP Jet Fusion 3D 4200 printer at 70 μm. The 1200 dpi print resolution in the XY-plane produces a spatial resolution of 0.317 mm × 0.228 mm in the build platform plane [18]. The frames were created in “Balance mode”. In this setting, all properties (speed, mechanical properties, dimensional accuracy, look and feel, agent efficiency) are at a balanced intermediate level, providing a good compromise between agent efficiency and mechanical properties [19].

The two large guide points for the biopsy needles are positioned in the same way for all frames: parallel to each other and at an angle of between 20° and 30° to the build platform. The two fixing points, which are almost parallel to the guide points, are fitted pointing downward. Figure 5 shows the alignment from an example print with seven frames. Since multiple frames were made at the same time, they are tightly packed on the XY-plane (Figure 5 top view). The printing time for a frame depends on the positioning in the build room and the associated build height. For the current study, the production of a frame, including the cooling phase and depowdering, took about 10–12 h on average (scaled by the entire build job). The subsequent cooling phase took place under room temperature conditions.

### 2.3. Validation and Measuring System

**Figure 6:**
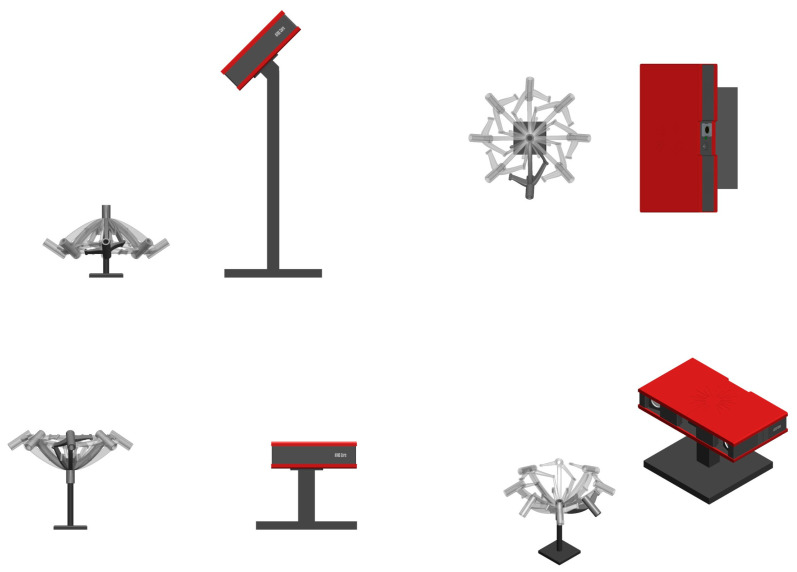
Measurement setup for accuracy evaluation of patient-specific frames with GOM ATOS Core 300 scanner.

To assess the technical accuracy of patient-specific stereotactic frames, an ATOS Core 300 optical 3D scanner (Carl Zeiss GOM Metrology GmbH, Braunschweig, Germany) was used. The volume to be measured was 300 × 230 × 230 mm^3^ with a measurement resolution of 5 MP. The measurement setup is shown in Figure 6. The frame was rotatably mounted on a rod via a biopsy needle guide point and rotated around its own axis in increments of approx. 45° to 50°. The camera was placed in two positions in relation to the frame (45° and horizontal), and an image was captured from each camera and frame position using the GOM Scan program (Carl Zeiss GOM Metrology GmbH, Braunschweig, Germany). Reference points (diameter 1.5 mm) on the object served to identify and reference the images to each other. Image data from individual camera positions were recorded, which were required to record the frames as a three-dimensional model. However, application of the strip light projection method meant that undercuts could not be recorded. As a result, some of the surfaces could not be fully reproduced.

Similarly, the through holes of the fixing points and guide points for the biopsy needles could not be sufficiently identified. Since the alignment of the frames is based on the five axes of the fixing and guide points, these axes were determined in a second measurement. For this purpose, the frames were also provided with five bolts of excess length, an interference fit in the holes, and then scanned. The bolts protruded between 20 and 30 mm on each side of the fixing or guide point, and the interference fit ensured a tight fit in the duct. In the next scan run, care was taken to ensure that the sheath surfaces of the bolts were captured sufficiently. A best-fit cylinder on the surface of the bolts made it possible to draw mathematical conclusions about the axis of travel of the through hole. In each measurement, the captured surrounding objects not belonging to the frame were removed, and the frames with and without bolts were subsequently adjusted. Polygonization produced an excellent quality STL file. All subsequent measurements were carried out with this experimental setup according to the same procedure.

### 2.4. Measuring System Suitability

To determine the suitability of the measuring system, the GOM ATOS Core 300 measuring system was examined on the basis of VDA 5 “Quality Management in the Automotive Industry, Process suitability”. This assessment involved carrying out a detailed analysis of the influencing components “test object” and “user”. The environmental influence (lighting and humidity) was assumed to be standardized and constant under laboratory conditions. The measurements were taken in an air-conditioned laboratory to ensure that both the frames to be tested and the measuring system were exposed to an ambient temperature of 22 °C.

To determine the standard uncertainty of the test object, one operator (user 1) took 25 repeated measurements taken with and without bolts. The standard operator uncertainty was determined based on three users with ten repeat measurements each on the same frame. Users 2 and 3 received detailed instructions on how to use the measuring system and were able to take the measurements independently according to the sequence protocol. The 30 measurements were taken in a randomized sequence. The purpose of measuring system suitability was to evaluate the systematic error produced by the GOM ATOS Core 300 measuring system used to measure patient-specific stereotactic frames.

**Figure 7:**
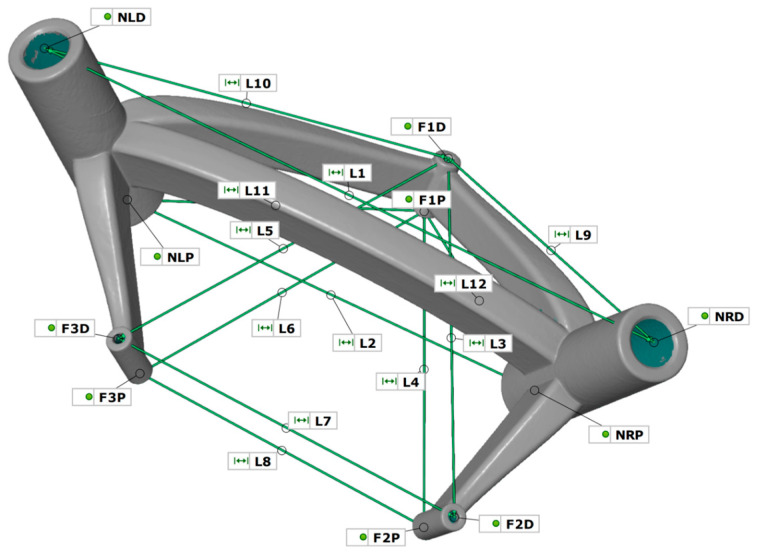
Measuring point definition for the measuring system suitability.

The models were prepared and evaluated in the GOM Inspect program. The lengths between the through hole axes were used as measurement parameters. In addition to determining the surfaces of the cylinder bolts, planes were determined on the end faces of the through holes with the same fitting method. The intersections of the five axes with the ten planes provided the measuring points for determining the length. The lengths are presented in Table 2 and Figure 7.

The measuring points were selected in such a way as to detect any possible change in the shape and position of the cylinders (three fixing points and two guide points). The four constructed triangles:

- Δ (F1D, F2D, F3D)
- Δ (F1P, F2P, F3P)
- Δ (F1D, NLD, NRD)
- Δ (F1P, NLP, NRP)

enable any imbalance and displacement of cylinder axes to be detected within and between the two components.

### 2.5. Target Point Comparison

**Figure 8:**
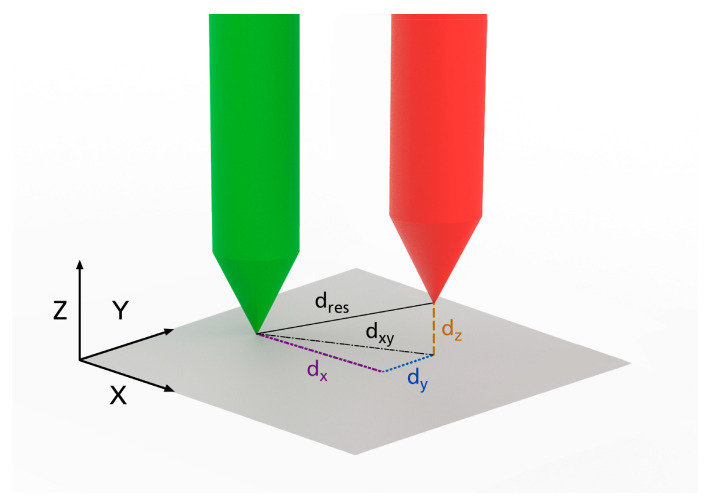
A schematic representation for determining the target point deviation. Left green needle point represents the planned target point and the right red needle point represents the measured actual target point. From the space coordinates, the distances $d_{xy}$, $d_{z}$ and $d_{res}$ determined.

All 16 frames were scanned using the measurement setup (Figure 6) with and without bolts and analyzed in GOM Inspect. The scan model was compared with the CAD model to determine the target point deviation. First, the scan frame was aligned with the CAD frame in a best-fit manner. To provide evidence of the target point deviation, the scan model was then registered with a reference point system (RPS) alignment to the CAD model. Compared to the best-fit, only defined points which were based on predefined reference points in the coordinate system were used for alignment. Points F1D, F2D and F3D, as well as F1P, F2P and F3P (Figure 7), were aligned to the corresponding CAD design points, and the quadratic error was minimized across all six points. This allows for the establishment of a realistic attachment to the skull via the bone anchors. By specifying the biopsy needle depth—determined from the design—an actual target point is constructed as an offset point over the axis of travel of the guide points and points NRD or NLD on the scan model. After the alignment of the scan model, this point is the virtual actual target point and describes the deviation of the target accuracy with the position in relation to the desired target point. The task of determining the measurement to the planned needle point was carried out in the three spatial directions *x, y* and *z*. The coordinate system was set up in such a way that the *xy*-plane was orthogonal to the needle axis *z* (Figure 8). According to Formula (1), from the target needle point, the deviation in the plane was determined as being $d_{xy}$ and the height offset as $d_{z}$. According to Formula (2), the resulting deviation $d_{res}$ is considered to be the absolute distance between both needle points.

(1) $$XY-Plane\;(d_{xy})=\sqrt{{\left(x_{expected}-x_{actual}\right)}^{2}+{(y}_{expected}-y_{actual})²}$$

(2) $$result\;distance\;(d_{res})=\sqrt{\;{\left(x_{expected}-x_{actual}\right)}^{2}+{(y}_{expected}-y_{actual})²+{\left(z_{expected}-z_{actual}\right)}^{2}}$$

To determine the variables influencing the target accuracy, two parameters were examined for accuracy. The first was the size of the frame: this involved calculating a bounding box for each frame, which delimits the frames in a right-angled box, with minimal edge lengths. The other variable was the biopsy needle depth, which indicates how far below the frame the target point is.

### 2.6. Sterilization

The geometrical distortion of the additively manufactured stereotactic frames was investigated in an autoclave that uses a steam sterilization process. The Systec DX-65 autoclave (Systec, Wetternberg, Germany) was employed for this. The frames were exposed to a temperature of 134 °C at a vacuum of 2–3 bar for a period of about one hour (machine program 2) [20,21]. The exposure to heat serves to survey the frames for possible material distortion. This process was performed once for each frame before the frames were digitally scanned again. The frames were digitized and measured as described in the earlier section.

### 2.7. Numerical Simulation

**Figure 9:**
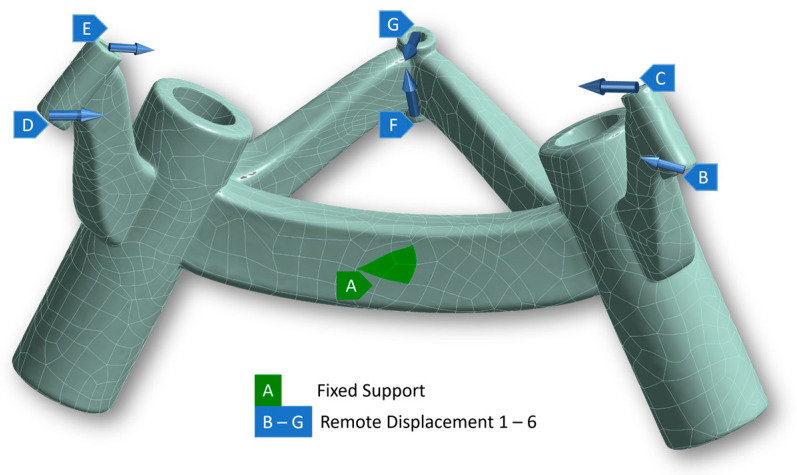
Restrictions for deformation analysis using the example of the small frame. With “A” as fixed support and “B–G” as remote displacement.

The smallest and largest constructed biopsy frames from the examination series were deformed in a numerical simulation in such a way that the fixing points were pulled or deformed to match the planned coordinate points that were envisaged when the patient-specific frames were designed. The scanned print models of the frames were available in STL format, and the Geomagic Freeform CAD 2020 program (3D Systems GmbH, Moerfelden-Walldorf, Germany) was implemented to convert the shell bodies into solids and save them in STEP format. Based on the STEP files, two FE models were created in Ansys Mechanical 2021 (Ansys Inc., Canonsburg, PA, USA). Three-dimensional tetrahedral elements (SOLID187) were generated for crosslinking. The inner surfaces of each of the three fixing points have two displacement boundary conditions (Figure 9). The structure was fixed to one face of the middle connecting arm by means of a boundary condition for clamping in the space.

This method allows the frames to be simulated and deformed in the same way as ideally happens on the patient’s head when scanning is conducted.

### 2.8. Statistics and Data Analysis

Statistical analysis of the data was conducted using SPSS (Version 24.0, IBM, New York, NY, USA). The Kolmogorov–Smirnov test was carried out to compute the normal distribution of the measurement system, whereas the Wilcoxon test was used to analyze non-normally distributed data in the case of target point deviation. For non-normally distributed data, when analyzing the target point deviation, the Wilcoxon test was used. *p*-values of 0.05 or less were considered statistically significant and *p* < 0.01 highly significant.

The data was processed and graphically displayed using the OriginPro (version 2023, OriginLab, Northampton, MA, USA) program.

## 3. Results

### 3.1. Measuring Accuracy of the ATOS Core 300 Measuring System

**Figure 10:**
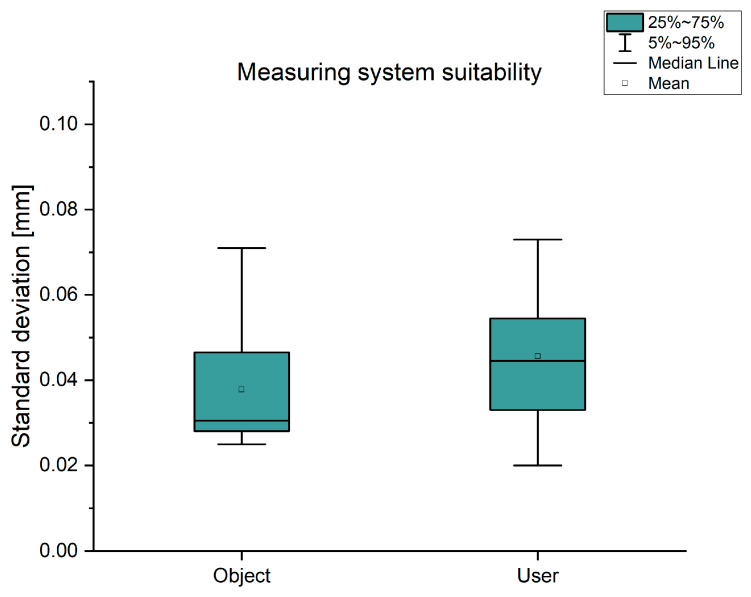
Measuring system suitability; object and user influence.

To evaluate the measuring system’s suitability, the influence of the measuring error from the object and user was determined. Ten measuring points (Figure 7) were determined in each measurement, from which the length measures L1 to L12 were calculated. Using the 25 measurements for the object influence and ten measurements per user for the user influence, the mean value was determined for each length, which in turn was formed using all twelve lengths. The standard deviations of the resulting mean values for the object and user are presented in Figure 10. The measurements from the object influence give a value of (0.04 ± 0.02) mm. By comparison, the 36 measurements of user influence are slightly higher at (0.05 ± 0.02) mm. This yields an average difference between measuring the object influence and measuring the user influence of 0.01 mm.

### 3.2. Target Point Deviation

**Figure 11:**
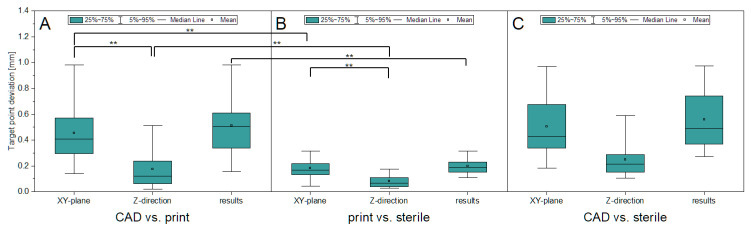
Target point deviation in the XY-plane, Z-direction and results in the comparisons CAD vs. print (**A**), print vs. sterile (**B**) and CAD vs. sterile (**C**). ** high significantly (*p* < 0.01).

Figure 11 presents a summary of the deviations for all 32 target points—divided into the XY-plane, Z-direction and the resulting deviation—of the frames in the CAD vs. print, print vs. sterile and CAD vs. sterile comparisons. The target point deviation between designed and printed frames (CAD vs. print) is significantly higher (*p* < 0.01) than the target point deviation of the printed frame after sterilization (print vs. sterile). The values given in the XY-plane are 0.16 mm ± 53.1% and 0.46 mm ± 51.9%. In the Z-direction, the deviations are 0.06 mm ± 81.4% and 0.17 mm ± 90.9%, and in the resulting deviation 0.18 mm ± 45.8% and 0.51 mm ± 48.3%. While a difference in the comparison between CAD vs. print and CAD vs. sterile exists, the samples do not differ at a significant level (*p* > 0.4). Within a group, the target point deviation in the Z-direction is always smaller than in the XY-plane (*p* < 0.01).

The target accuracy results are presented in Table 3 using the mean value and standard deviation.

### 3.3. Correlation of Frame Size

**Figure 12:**
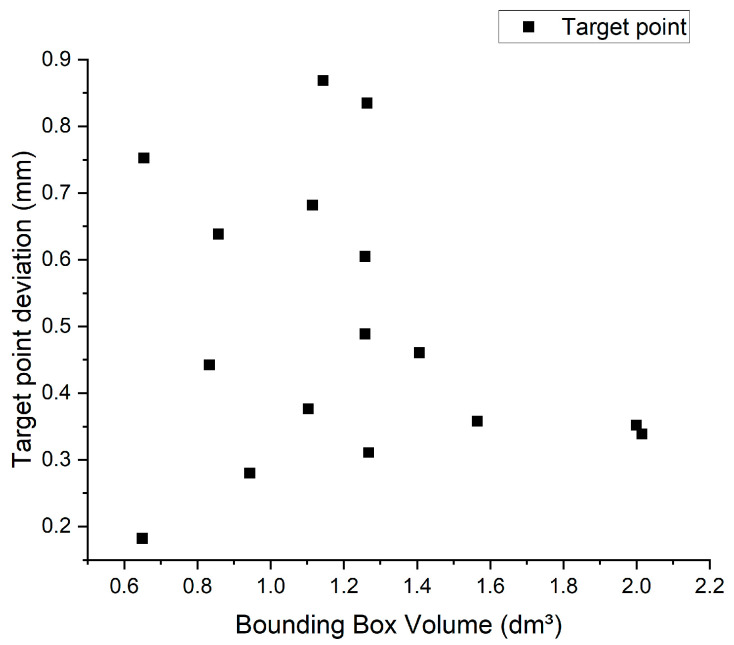
Correlation between frame size and target point deviation in CAD vs. sterile comparison as mean value right and left target point.

The volume of the designed frame is represented in Figure 12 as a bounding box in relation to the target point deviation. The biopsy frame sizes range from 0.65 dm^3^ to 2.01 dm^3^, corresponding to a size increase of 309%. The values shown in Figure 12 are the mean values for the sterilized biopsy frames from a right and left target point deviation. The smallest deviation was achieved by biopsy frame No. 6 (0.65 dm^3^) with 0.18 mm and the largest deviation by frame No. 10 (1.14 dm^3^) with 0.87 mm.

### 3.4. Correlation of Needle Depth

**Figure 13:**
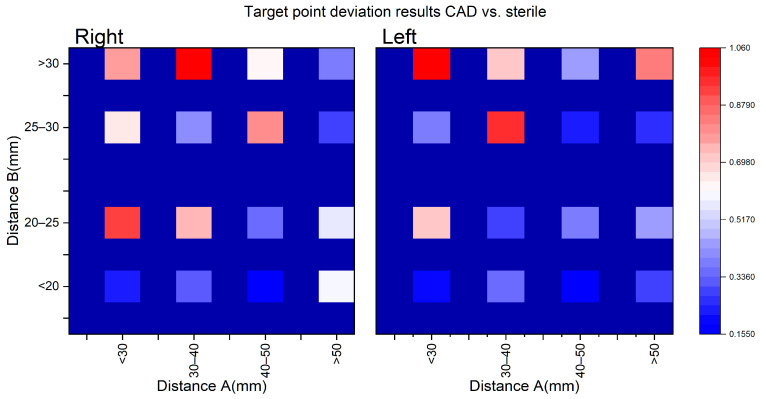
Influence of distance A and B of the target point deviation of stereotaxic frames after sterilization (CAD vs. sterile).

In the case of the second influencing parameter, the correlation between the target point deviation and needle depth was investigated. The needle depth was the sum of distance A (target point to skull) and distance B (skull to frame). In Figure 13, the resulting deviation values of the sterilized biopsy frames (CAD vs. sterile) for the right and left target points are presented as a heat map. It shows that the target point deviations are smallest with the smallest distance B. As distance B increases, the accuracy of the biopsy frames decreases. No difference in behavior was established between the right and left target points. As distance A increases, no increase in the target point deviation was found to be evident.

The range from the closest point to the skull and the deepest target point in the brain (distance A) extends from 22.41 mm to 72.39 mm. Distance B ranges from 16.07 mm to 39.48 mm, producing biopsy needle lengths of 42.33 mm to 97.90 mm for the 32 target points.

### 3.5. Target Point Deviation after Numerical Simulation

**Figure 14:**
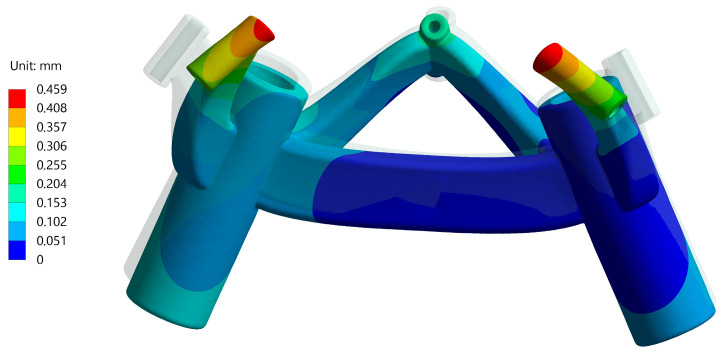
Deformation result from the simulation with 40 times magnification on the example of the smallest frame No. 6.

Frame No. 6, being the smallest biopsy frame, was examined in more detail, as was No. 14 as the largest. The deformation results for frame No. 6 are presented with a magnification scale of 40 in Figure 14. The adjusted displacement of the fixing points is transferred to the connecting arms and causes a slight change in the position of the guide points.

**Figure 15:**
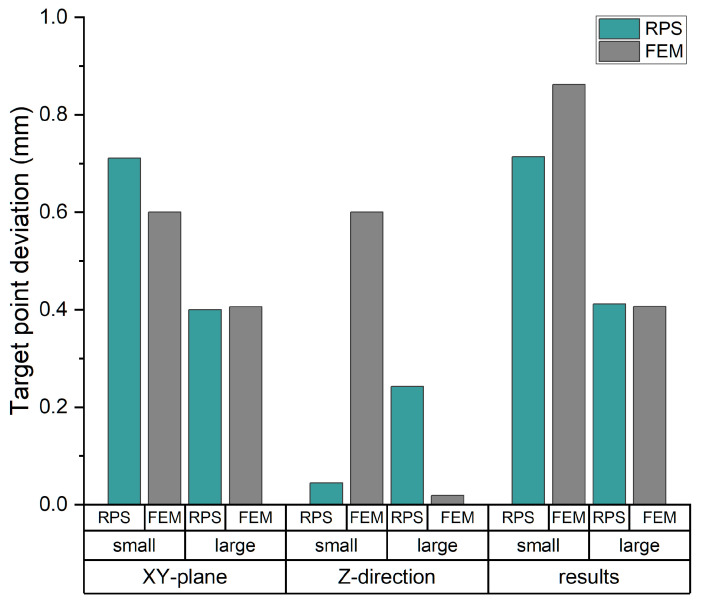
Target point deviation of the largest and smallest stereotaxis frame after RPS alignment and numerical simulation (FEM).

The impact of the modified guide points was analyzed as follows. Figure 15 shows the deviations detected between the two methods used—RPS alignment using GOM Inspect and the finite element method in Ansys. The deviations in the XY-plane, Z-direction and the resulting deviation are differentiated according to the frame size and method. The mean value for each frame is considered from the right and left target points.

For the smallest biopsy frame, a higher deviation of the target point was detected in the XY-plane with the RPS alignment than with the FEM analysis (Δ0.11 mm). The results were almost identical for the largest frame (Δ0.01 mm). In the Z-direction, the results were reversed for the small frame. In this instance, the FEM analysis results produce high target point deviations (Δ0.56 mm). In the case of the large frame, the deviation in the axis is close to zero based on the FEM analysis, resulting in a difference of 0.22 mm. When considering the resulting deviation, both methods achieve similar values (Δ0.01 mm) for the largest frame and higher deviations (Δ0.15 mm) for the smallest biopsy frame based on the FEM analysis. The results for all the comparisons are listed in Table 4.

## 4. Discussion

Biopsies are carried out unilaterally and serially. In a clinical context, this means that there is one guide point for the biopsy needle per frame. Therefore, tissue is collected at several heights along the planned trajectory. The experimental setup the patient-specific stereotactic frames with two biopsy target areas allows the target point analysis to be doubled in the 16 frames designed and studied. The geometry of the frames has been proven in clinical studies [22,23] and has been continued in this way. In contrast to Gutmann et al. 2020, the dimensions of the two guide points and three fixing points have remained the same. However, the cross-sectional area of the connecting arms on the guide points was increased by 10 mm^2^ for enhanced stability.

### 4.1. Additive Manufacturing

When selecting materials and processes with PA12, care was taken to ensure that the material was biocompatible and therefore also a viable candidate for future application in stereotactic frames. In combination with Hewlett Packard’s Multi Jet Fusion manufacturing process, which focuses on high resolution and accuracy of the components, it is shows considerable promise for meeting the requirements of a high-precision medical navigation system used to perform a brain biopsy.

In additive manufacturing technology, the quality of a component depends on many factors and influencing variables. Rehme 2010 provides an overview and guidelines that show the influence of certain aspects and presents them in an Ishikawa diagram [24]. Print setting is one user-dependent parameter. “Balance Mode” enables the quantitative and reproducible programming of the defined machine-related settings. However, the alignment of the frames in the build room is a limitation of the study. Based on empirical values, the frames were positioned with guide points inclined at an angle of 20–30° to the build platform, which is not a fixed size, especially since the right and left needle guide points do not run in parallel. Similarly, the different frame sizes made standardized positioning difficult. The influence of a different position in the build room was not investigated within the scope of this study.

### 4.2. Study Design

To exclude the influencing variables from clinical CT or MRI imaging (slice height, field of view, matrix, intensity) when verifying target points, an alternative method to the in vivo study was used. As described in the measurement setup, strip light projection technology [25,26] was used to digitize the frames after printing and sterilization. The benefit of these methods is that high-precision metrology has been used for evaluating the distortion of the frames after additive manufacturing and the sterilization process, with a finer measurement resolution than clinical imaging systems (CT, MRI). The resolution of the MRI used for target point planning has a 1 mm slice thickness and 0.4 mm × 0.4 mm pixel size in the plane. On the other hand, the GOM ATOS Core 300 has a measuring field of 300 mm × 230 mm with 5 megapixels. With a technical resolution of 2452 × 2056 pixels, the technical resolution for the measuring point distance is 0.12 mm. This value is corrected by internal calculation processes, so that a length measurement deviation of 0.022 mm can be specified after calibration. Projection onto the plane means that it is three times more accurate than clinical imaging (0.4 vs. 0.12). The downside of this method is that strip light projection is limited to visual surfaces. However, since the hole diameters of the three fixing points and two needle guide points play a crucial role in determining accuracy, the method was modified with bolts inserted in the holes. The suitability of the measuring system was assessed on the basis of VDA 5 “Quality Management in the Automotive Industry, Process suitability,” which sets a very high standard. The medical requirement of the entire system of the stereotactic device is less than 2 mm. According to the definition of the golden rule in metrology (measurement uncertainty maximum 1/10 of tolerance), [27] the measuring system may present inaccuracies of up to 0.2 mm; the findings presented here were considerably lower, at four times below this value (0.046 mm ± 0.016 mm), when measurements were taken using the GOM ATOS Core. The results of the user and object influences regarding errors demonstrate the potential of this measuring system for future quality assurance of patient-specific stereotactic frames, which have five guide cylinders including five connecting arms. The average difference of 0.01 mm between the user and object is so small that it is negligible when considering the accuracy of stereotactic frames and is below the system’s measurement accuracy.

The limitation in terms of detecting non-visible surfaces (holes) has been shown to be acceptable and not detrimental with the extended bolt method.

### 4.3. Numerical Simulation

The results of the numerical simulation corroborate the RPS alignment method. The results of the finite element method show that the target point deviation for the smallest and largest frames is less than 1 mm and thus below the required 2 mm limit. By carrying out an additional simulated deformation of the frames in the direction of the specified mounting points on the skull, no larger target point deviations are achieved than with the RPS method used here. However, there is one striking fact, which is that the simulated deformation in the Z-direction is more significant for small frames. This is probably due to the fact that the connecting arms of the small frames are shorter compared to those of a large frame, and a displacement of the fixing points is transmitted more directly to the needle guide points. A longer connecting arm entails a larger level and the deformation of the connecting arms can be spread over a greater distance due to their flexibility.

### 4.4. Target Point Deviation of the Patient-Specific Frame

The results of the target point deviation, compared to printing, show a significantly smaller deviation of the target points due to sterilization. Conversely, this means that the additive manufacturing process results in the largest deviations from the target geometry. However, the deviation of the XY-plane is 0.46 mm ± 0.24 mm, which is significantly lower than the required accuracy of 2 mm. When looking at the Z-direction in isolation in the CAD vs. print comparison, target points are 2.5 times more accurate. If both processes (manufacturing and sterilization) are considered together, the target point deviation resulting from the XY-plane and the Z-direction is 0.50 mm ± 0.24 mm, which means that the limit is four times below the permissible deviation. One explanation as to why the CAD vs. sterile comparison does not result in the cumulative results from CAD vs. print and print vs. sterile is that they cancel out the values at 32 target points or that the sterilization process may have a positive effect on the target accuracy. In the print vs. sterile comparison, only the deviations of the sterilized frames to the printed frame are listed. However, the target point can also shift in the positive direction, towards the planned coordinates of the target point, which is not covered in this comparison. Taking a close look at the data shows that the target point in the XY-plane is improved by 0.02 mm, in the Z-direction by 0.01 mm and in the resulting by 0.02 mm in the CAD vs. sterile comparison after printing. However, about half of the samples tend to have a more accurate target point, while the other half tend to have an increased target point deviation. A systematic effect similar to that of heat treatment for metals is not apparent.

Given the results, the examined frame size does not appear to play a major role when considering the accuracy of the needle position. Biopsy frames were created from smallest to largest in a range of 1.36 dm^3^. The frame size was determined by the position of the bone anchors in the skull and the distance to the skull. Target point accuracy does not correlate with frame size, nor does frame alignment, regardless of whether the triangle comprising F1, F2 and F3 (Figure 7) is aligned forward or backward (Figure 4 shows a forward-facing triangle). The analysis of the numerical simulation underscores what needs to be considered in the case of the bone anchor placement and resulting frame size. Small frames tend to produce a larger target point deviation in the Z-direction than frames with larger connecting arms. However, this trend is not evident in any comparison of RPS-aligned frames.

On the other hand, needle depth shows an influence on target accuracy, although there is no clear correlation. For a more detailed view, the needle depth was divided into distance A and B and the dependencies were examined. The results highlight the fact that distance A does not increase or decrease the deviation of the biopsy needle with increasing length (<30 mm to >50 mm). Figure 13 illustrates a homogeneous color distribution in the matrix rows. The best results can be achieved with distance B below 20 mm. During the design of the biopsy frames, the minimum distance for B was 16.07 mm. Seven of the eight target points examined were at the B < 20 mm setting with an accuracy of less than 0.34 mm. In comparison, the smallest deviation was in the B > 30 mm setting with 0.38 mm at a distance B of 35.95 mm. Among the B distances < 20 mm and >30 mm, there is an average difference of 0.41 mm. There is no statistical correlation between the resulting length from distance A and B and the target point deviation in the Z-direction for the CAD vs. sterile comparison. In summary, the target point deviation tends to increase with larger B values, whereas this is not observed with increasing A values. 

### 4.5. Comparison with Available Patient-Specific Neurosurgical Stereotactic Frames

The target point deviations shown in this study are best compared to those resulting from the patient-specific stereotactic platform STarFix system studied by Konrad and colleagues [10]. Similar to the STarFix system, the biopsy frame in the current study is designed custom to the individual patient and produced using additive manufacturing processes. In contrast to the STarFix system, the design data are MRI-based [12] and do not require X-ray-based computerized tomography. Similarly, the material (PA12) with a heat deflection temperature of 175 °C at 0.45 MPa [16] is designed for steam sterilization. In vivo studies show an accuracy from 2.0 mm ± 0.9 mm [10] up to 2.8 mm ± 0.84 mm [28]. According to an in vitro study, the accuracy increases to 0.42 mm ± 0.15 mm at 31 virtual target points on a model [29].

### 4.6. Comparison with Available Conventional Neurosurgical Stereotactic Frames

Here, the collected results are classified in comparison to other stereotactic systems. Frame-based stereotactic systems were conventionally considered to be the most accurate systems used in stereotaxy. The Leksell G-Frame (Elekta, Stockholm, Sweden) shows an electrode deviation of 1.2 mm ± 0.6 mm [2] and 1.5 mm ± 0.8 mm, respectively, in studies when placing needle electrodes for deep brain stimulation [30]. In follow-up studies comparing frame-based, frameless, and robot-based systems, the accuracy for the frame-based systems is of a similar order of magnitude at 1.93 mm [31] and 1.11 mm ± 0.59 mm [32]. Frameless systems, however, lag more than twice as far behind with an accuracy of 2.89 mm [31] and 2.96 mm ± 1.49 mm [33]. However, Kesserwan and colleagues were unable to establish any difference in diagnostic yield between frame-based and frameless systems in more than 3000 patients [34]. Robot-based stereotactic systems show the widest scatter range from 0.76 mm ± 0.37 mm [32] to 1.95 mm ± 1.11 mm [35], but also the smallest target point deviations. This shows the potential of robotic stereotactic systems compared to frame-based and frameless systems in terms of accuracy, precision and operation time analyzed [32].

### 4.7. Limitation of the Study and Other Variables Influencing

The aim of the present study method was to investigate the influence of additive manufacturing technology and the steam sterilization process on the accuracy of patient-specific stereotactic frames made from plastic. The procedure allows an assessment of the technical accuracies of these two variables influencing the overall accuracy of a biopsy from designing the frame to placing it on the patient’s head. Consequential influences, such as fixing the frame to the patient’s skull using bone anchors, cannot be investigated with these study designs. This involves adding boundary conditions such as the behavior of the bone anchors in the skull during fixation. On the one hand, the strength of the bone anchors in the skull plays a role in clinical use and, on the other hand, so does the way in which the bone anchors in the skull may shift if the fixing points of the frames misalign with the screw-in points of the bone anchors. In terms of the design and ultimately the technical accuracy of the stereotactic frames, the strength of the bone anchors in the skull bone is highly relevant. If the bone anchors were to come loose or shift between placement and attachment of the frames, this would jeopardize an accurate biopsy. This process is not considered and must be examined in future studies. Likewise, the influence of a brain shift fell outside the scope of this study. Brain shift describes the movement and deformation of the brain in relation to its anatomical and physiological position in the skull. These shifts can have a significant impact on accurate biopsy retrieval and showed individual aspects of brain shift in stereotactic procedures and in the implantation of electrodes for deep brain stimulation have been investigated in the past [36,37,38].

## 5. Conclusions

The investigated technical accuracy in relation to the influence of the manufacturing process and the sterilization process for the patient-specific stereotactic frames has proven suitable and sufficiently accurate for performing a biopsy. On average, the results of the 32 target positions examined are four times below the clinically required accuracy of 2 mm for a biopsy. The patient-specific stereotactic frames, which were manufactured using HP’s Multi Jet Fusion additive process with the material PA12, meet the requirements of modern neurosurgical navigation and do not make any compromises when it comes to accuracy. In addition, the material is suitable for steam sterilization due to its barely noticeable distortion. The use of patient-specific stereotactic frames is a time- and cost-effective alternative to conventional methods. The process chain used to create and manufacture patient-specific frames has proven to be tried and tested and shows promise for growing clinical use in future. Future expansions to the study conducted here involve an in vitro study, which is planned to investigate the clinical target point deviation on the preparation. Eliminating the use of computerized tomography represents a major benefit over conventional systems. In addition to stereotactic navigation of deep brain stimulation, the consistently excellent achievement of target accuracies will enable an array of neurosurgical interventions in the future. For example, the patient-specific stereotactic frames presented here can also be used in interstitial laser thermal ablations [39,40] and brainstem hemorrhages [41].

## Figures and Tables

**Table 1:** Technical parameters for MRI imaging.

Parameter	3-Tesla MRT
Sequence	T1W
Cutting plane	sagittal
Coil	head
Field of View	260 mm
Repetition time	9.3
Echo time	4.3
Matrix	640
Slice thickness	1 mm
Pixel size	0.4 mm × 0.4 mm

**Table 2:** Length designation for measuring system suitability.

**L1**	**NRD** Needle guide point right distal	↔	**NLD** Needle guide point left distal
**L2**	**NRP** Needle guide point right proximal	↔	**NLP** Needle guide point left proximal
**L3**	**F1D** Fixing point 1 distal	↔	**F2D** Fixing point 2 distal
**L4**	**F1P** Fixing point 1 proximal	↔	**F2P** Fixing point 2 proximal
**L5**	**F1D** Fixing point 1 distal	↔	**F3D** Fixing point 3 distal
**L6**	**F1P** Fixing point 1 proximal	↔	**F3P** Fixing point 3 proximal
**L7**	**F2D** Fixing point 2 distal	↔	**F3D** Fixing point 3 distal
**L8**	**F2P** Fixing point 2 proximal	↔	**F3P** Fixing point 3 proximal
**L9**	**F1D** Fixing point 1 distal	↔	**NRD** Needle guide point right distal
**L10**	**F1D** Fixing point 1 distal	↔	**NLD** Needle guide point left distal
**L11**	**F1P** Fixing point 1 proximal	↔	**NLP** Needle guide point left proximal
**L12**	**F1P** Fixing point 1 proximal	↔	**NRP** Needle guide point right proximal

**Table 3:** Values of the target point deviation in the XY-plane, Z-direction and results in the comparisons CAD vs. print, print vs. sterile and CAD vs. sterile.

	CAD vs. Print		Print vs. Sterile		CAD vs. Sterile	
	Mean	STD	Mean	STD	Mean	STD
XY-plane	0.46	0.24	0.16	0.05	0.44	0.18
Z-direction	0.17	0.16	0.06	0.04	0.17	0.11
results	0.51	0.23	0.18	0.05	0.50	0.20

**Table 4:** Values of the target point deviation of the largest and smallest stereotaxis frame after RPS alignment and numerical simulation (FEM).

	RPS		FEM		Difference	
	Small	Large	Small	Large	Small	Large
XY-plane	0.71	0.40	0.60	0.41	−0.11	0.01
Z-direction	0.05	0.24	0.60	0.02	0.56	−0.22
results	0.71	0.41	0.86	0.41	0.15	−0.01

## Data Availability

The data presented in this study are available on request from the corresponding author. The data are not publicly available due to privacy restrictions.
